# The Need for Equitable Scholarship Criteria for Part-Time Students

**DOI:** 10.1007/s10755-021-09549-7

**Published:** 2021-04-19

**Authors:** Alexandria N. Ardissone, Sebastian Galindo, Allen F. Wysocki, Eric W. Triplett, Jennifer C. Drew

**Affiliations:** 1 Microbiology and Cell Science Department, College of Agricultural and Life Sciences, Institute of Food and Agricultural Sciences, University of Florida, Gainesville, FL 32611 USA; 2 Agricultural Education and Communication Department, College of Agricultural and Life Sciences, Institute of Food and Agricultural Sciences, University of Florida, Gainesville, FL 32611 USA; 3 Office of the Dean, College of Agricultural and Life Sciences, Institute of Food and Agricultural Sciences, University of Florida, Gainesville, FL 32611 USA

**Keywords:** Part-time, Transfer students, Online education, Nontraditional students, Scholarships, Equity

## Abstract

Current policies and interventions to enhance student success and retention are often tied to full-time enrollment, which are substantiated by studies associating part-time enrollment with lower retention and poorer academic outcomes. However, these findings are limited to studies of first-time college students and do not represent today’s nontraditional undergraduate who may be transfer, online, and working adult students. To enhance retention of on-campus *and* hybrid online 2 + 2 transfer students in a STEM undergraduate program, need-based scholarships are awarded to students enrolled full-time as stipulated by the federal granting agency. Although the scholarship has helped >80 students to date, over 40% of eligible transfer students are excluded only because they are enrolled part-time, disproportionately affecting students in the hybrid online track who are more likely to be women and underrepresented minorities. Using quantitative and qualitative methods, this study explores enrollment behavior of transfer students (online and on-campus), its relationship with student characteristics and academic outcomes, and implications for scholarship eligibility. Full-time enrollment is a significant challenge for transfer students. While scholarship requirements are a key factor influencing full-time enrollment, online transfer students additionally cite work and family obligations as drivers of enrollment behavior. Thus, online 2 + 2 transfer students face different challenges than on-campus peers and are more likely to enroll part-time out of necessity, yet still have comparable GPA and graduation rates. These findings support a growing awareness of barriers nontraditional students face questioning the relevance of policies driven by “traditional” student outcomes, which perpetuate inequity in higher education.

**Supplementary Information:**

The online version contains supplementary material available at 10.1007/s10755-021-09549-7.

## Introduction

Sparked by the national call to enhance science, technology, engineering, and mathematics (STEM) education in the United States (Holdren et al., 2013; National Academies, 2010; Olson & Riordan, 2012), a microbiology department at an AAU land-grant university developed a 2 + 2 hybrid online distance education track for transfer students in the A.A. to B.S. pathway in order to increase diversity and access to a STEM undergraduate degree (Drew et al., 2015). In this 2 + 2 program, students transfer from a 2-year institution and pursue a Bachelor of Science degree through online coursework and face-to-face bootcamp lab courses (Ardissone et al., 2019). The hybrid online 2 + 2 model was shown to have comparable student outcomes as the on-campus 2 + 2 program and addressed the equity gap in STEM by broadening participation of women and underrepresented minority students (URM) (Drew et al., 2016).

Although the online and on-campus transfer cohorts had comparable outcomes, their retention and GPAs were lower than on-campus students who started at the university and did not transfer (Drew et al., 2015; Drew et al., 2016). Generally speaking, transfer students in the 2 + 2 pathway are more likely to be low-income, from underrepresented minority backgrounds, female, financially independent, first generation students, veterans, and working parents as compared to their non-transfer peers (Labov, 2012; Radwin et al., 2013; Teacher Advisory Council, 2012). Transfer students have lower retention and graduation rates (Jenkins & Fink, 2016; Shapiro et al., 2013) than non-transfer students and these rates are even lower for transfer students from backgrounds traditionally underrepresented in STEM (Núñez & Elizondo, 2013; Reyes, 2011). Researchers have identified several challenges that affect the student transfer process and success. The challenges include factors such as financial and cultural barriers and disparities in academic preparation (Núñez & Elizondo, 2013; Senie, 2016; Shapiro et al., 2017).

In 2017, the program received a grant from the National Science Foundation focused on the unique paths and challenges of STEM transfer students with the goal of increasing retention and success through scholarships and other interventions. The majority of the award provides need-based scholarships to transfer students in the 2 + 2 pathway. As with many scholarship programs and tuition assistance programs, the grant stipulated full-time enrollment for recipients. It was quickly observed that many low-income students were ineligible to receive the scholarship only because they were enrolled part-time.

In 2016, 7.7 million students were enrolled part-time in institutions of higher education, and this number is projected to increase to 8.1 million by 2027 (Hussar & Bailey, 2019). Part-time enrollment is disproportionately distributed between 2- and 4-year institutions, with 62.1% and 28.6% students enrolled part-time, respectively (Hussar & Bailey, 2019). Students who enroll part-time are more likely to be older, female, Hispanic, lower income, and first-generation students compared to students who enroll exclusively full-time (Chen, 2007). Also, as implied by being older, part-time students are also more likely to be financially independent and work full-time, be married, and have one or more dependents. Nearly half (48%) of online students attend part-time and that part-time students are more likely to enroll in online classes (Chen et al., 2010; Ortagus, 2017; Venable, 2019). Taken together, it is expected that transfer students in the 2 + 2 track would be more affected by external factors than ‘traditional’ beginning undergraduate students because their characteristics align with conceptual models of nontraditional student attrition (Bean & Metzner, 1985).

Students who enroll less than full-time do not qualify for as many types of tuition assistance, and the financial burden of attending college is much greater than compared to full-time students (reviewed in Grabowski et al., 2016). The average amount of gift aid for full-time students is $11,200, while the average amount of gift aid for part-time students is just $4200 (Radwin et al., 2018). However, the reduction in aid is not proportional to the reduction in the cost of college for part-time students when the full scope of expenses (including books, supplies, housing, food, and childcare) is considered. Indeed, the average annual costs beyond tuition are statistically higher for part-time students compared to full-time students (Palacios et al., 2020).

Several studies report part-time enrollment is negatively associated with degree attainment and persistence (Berkner et al., 2003; Chen, 2007; O'Toole et al., 2003). However, these findings were based on data limited to beginning, or first-time in college, students who were younger than the typical part-time student. The heterogeneity of nontraditional student characteristics has made this group difficult to study, and this gap in knowledge has led to educational policies that often overlook a significant proportion of students enrolled in postsecondary education. The Integrated Postsecondary Education Data System’s (IPEDS) categorization of students by first-time enrollment and full/part-time enrollment status (first-time, full-time; first-time, part-time; non-first-time, full-time; and non-first-time, part-time students) is encouraging (Campbell & Bombardieri, 2017). Nevertheless, the view that part-time enrollment is negatively associated with graduation and persistence has endured in shaping national educational policy. Strategies to improve part-time students’ outcomes largely involved getting these students to enroll full-time (Bombardieri, 2018). However, implications of part-time enrollment on student outcomes is nuanced and distinction of additional factors beyond enrollment status should be considered. Due to the increased responsibilities often borne by older students’ life factors, many students enroll part-time out of necessity. There is a growing awareness that exclusively full-time enrollment is not the best for all students, and that part-time enrollment is the only feasible path to earn a degree for some students (Bombardieri, 2018; Fain, 2015).

The demographic, academic, and financial disparities characteristic of transfer students warrants the need for intervention strategies, such as scholarships, to promote academic success of transfer students. Yet, enrollment patterns of transfer students, particularly those in online programs, would have detrimental implications for the reach of scholarships, or any intervention, whose eligibility is conditioned on full-time enrollment. This study explores predictors, academic outcomes and student motivations of enrollment behavior amongst different student groups (first-time in college, on-campus transfer, and online transfer students) and challenges the legitimacy of full-time enrollment criteria as it is applied indiscriminately in scholarship policy.

## Materials and Methods

### Study Populations & Data Collection

This study describes the enrollment trends of upper-division microbiology majors at an AAU land-grant university from fall 2018 to spring 2020. Students were grouped by matriculation status and track into the following three groups: first-time in college (FTIC), on-campus transfer (OC-TR), and online transfer (ONL-TR) students. The microbiology department developed a 2 + 2 online transfer program in 2011 (Drew et al., 2015), which aimed to broaden participation and increase accessibility for underserved students aspiring to earn a B.S. degree in STEM. Students in the 2 + 2 online track complete their associate degree at another institution then transfer to the university as an online (remote) student. Hence, they are considered upper-division students upon transfer. Therefore, enrollment data considered in this study has been restricted to upper-division enrollment for all student groups. Transfer data is limited to those students in either the on-campus or online transfer 2 + 2 track and does not include transfers from other 4-year granting institutions. All courses are delivered online regardless of student group (with the exception of labs), and the majority are considered upper division courses, requiring the completion of general science courses as a prerequisite, which is satisfied in the first 2 years (either at the university or another institution for FTIC or transfer students, respectively).

With the intent of better understanding and facilitating the success of transfer students in microbiology, the department was awarded a grant to administer scholarships to eligible transfer students through a STEM scholarship program from a federal agency. Enrollment data were collected for fall and spring semesters from Fall 2018 to Spring 2020 (2 academic years, or 4 semesters of data) to coincide with the years in which the scholarship would have been available to microbiology majors. Non-citizens of the United States were excluded from analyses as this group comprised a small percentage (<8%) of the student population, the majority of which did not qualify as eligible for the award as stipulated by the funding agency. With these exclusion criteria, a total of 603 students were included in the data set for the quantitative phase of this study.

**Fig. 1:**
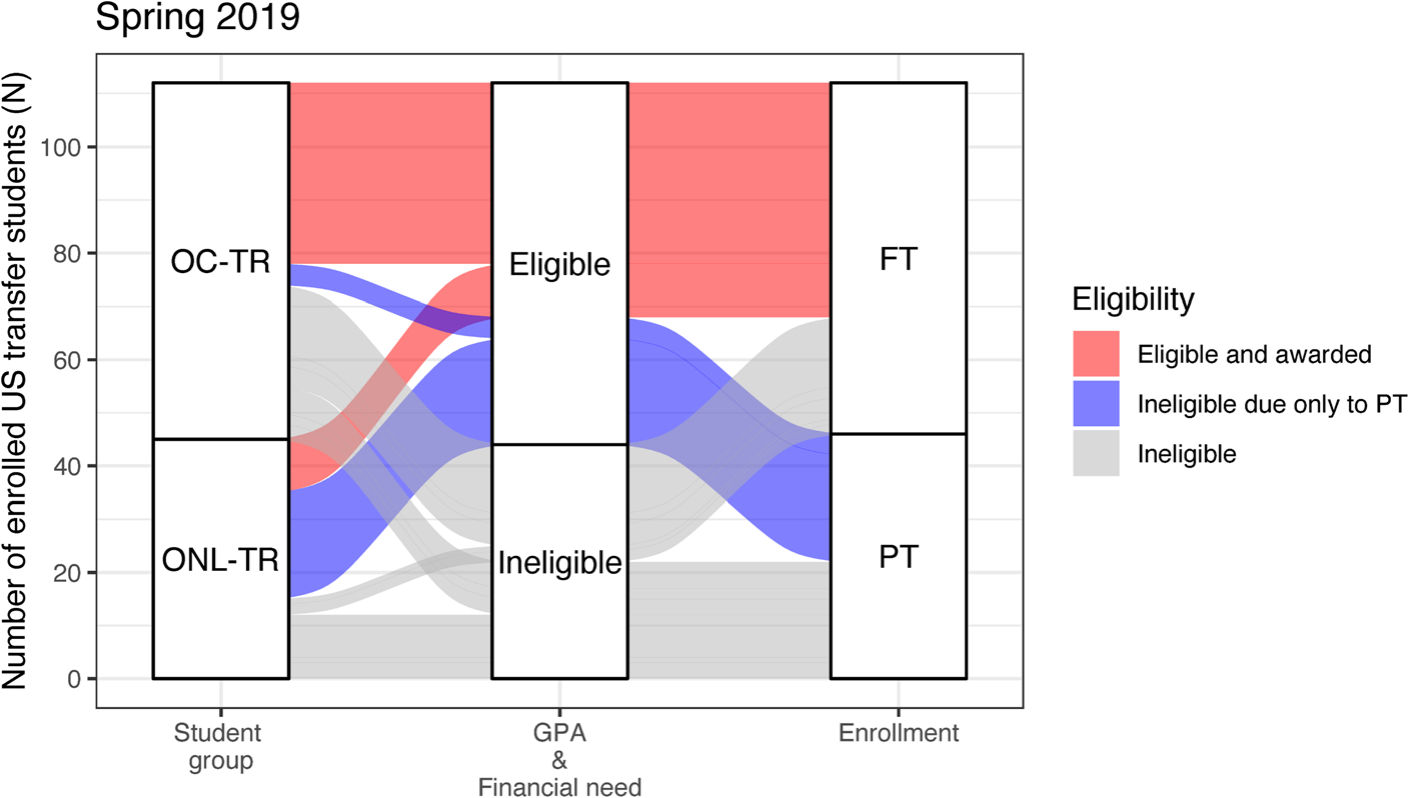
Alluvial plot to visualize the flow of scholarship eligibility of transfer students for the Spring 2019 term (*N* = 112). Students are represented by a “track” that flows through the eligibility criteria. The width of the track corresponds to the number of students who shared eligibility criteria. On-campus (OC-TR) and online (ONL-TR) transfer students are separated in the first bar column in order to illustrate how they differ in eligibility criteria (colors). Eligible and thus awarded students (*N* = 44) are tracked in light red. Students ineligible to receive the scholarship due only to PT enrollment are tracked in purple and distinguished in the third column. Students ineligible due to poor academic merit (GPA <2.5) and/or no or undetermined financial need are tracked in gray and differentiated in the second column. Only US transfer students were included in this analysis; while there were eligible non-citizens considered for the scholarship, this criterion is not easily discernible systematically and would violate student privacy rules

Retention data (4-year) were collected from microbiology major student cohorts that matriculated fall 2011 to summer 2014. Data from the spring 2019 semester was selected as a representative data set for analysis illustrated in Fig. 1 and Supplementary Table 1. Survey data were collected online via Qualtrics (Qualtrics, Provo, UT, 2019) fall 2019 and was administered to all microbiology majors, in which there were 71 respondents (15.2% response rate). The survey questionnaire consisted primarily of multiple-choice and Likert scale questions aimed at capturing attitudes, behaviors, aspirations, and demographic characteristics of respondents. Three open-ended questions were also included to better understand the reasoning behind enrollment attitude and behavior. Data were deidentified, and a minimum reporting threshold of 5 students was applied (denoted with asterisks (*) in tables). This study was approved as exempt by the university’s Institutional Review Board (IRB201601296).

### Data Analysis

Using de-identified enrollment data from fall 2018 to spring 2020, excluding summer semesters, descriptive statistical analyses comparing the three student groups, FTIC, OC-TR, and ONL-TR, were performed and significant differences (*p* value <0.05) determined using Fisher’s Exact test or Kruskal-Wallis test for categorical or numerical variables, respectively. Differences in student demographic variables were explored. Underrepresented minority (URM) students in STEM included Black, Hispanic, American Indian or Alaskan Native, Native Hawaiian or other Pacific Islander (Garrison, 2013). Student age was calculated at the time matriculation to the university. Course load and GPA were averaged for each student for all fall/spring semesters enrolled from fall 2018 to spring 2020, where students averaging less than 12 credits were deemed part-time. Eligibility for Pell federal funding was used as a proxy to determine student financial need and is an underestimation of true financial need.

**Fig. 2:**
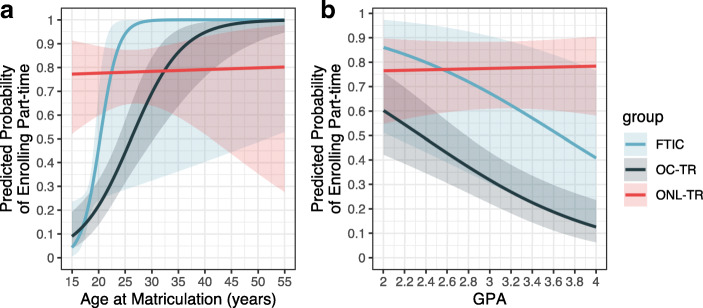
Predicted probabilities of enrolling part-time for various ages (**a**) or GPAs (**b**) across different student groups. Predicted probabilities were calculated for male, non-URM students with an average GPA (3.25) when varying values of age or average age (21 years) when varying values of GPA. Shaded regions represent 95% confidence intervals

Logistic regression was used to identify variables affecting part-time enrollment. The demographic and academic performance variables considered were sex, URM status, age, GPA and student group (FTIC, OC-TR, or ONL-TR). Based on descriptive analyses, interaction terms included in the model were: 1) student group by age and, 2) student group by GPA; both of which were significant. Coefficients and confidence intervals were exponentiated to calculate odds ratio and corresponding 95% confidence intervals (Table 2). Nonlinear transformation of the logistic regression results to predicted probabilities was used in order to interpret significant interactions (Fig. 2). All statistical tests and data visualizations were performed in R 3.6.0 (R Core Team, 2019). Responses to Likert style questions were translated to numeric values and centered at zero in order to perform statistical analyses and visualization.

Qualitative data from individual responses from open-ended questions were imported into the qualitative data analysis software NVivo (QSR International, 1999). Thematic analysis was performed within Nvivo applying a four-step process to analyze a conceptual theme (Harding, 2018). Data were coded during the process following published recommendations (Saldaña, 2015). Demographic classifiers, such as student group and type of enrollment, were connected as attributes to each participant.

## Results

### Distinguishing Characteristics of Online Transfer, on-Campus Transfer, and First-Time in College Students

Given the different set of challenges that transfer students encounter, a descriptive analysis of the three student groups in the microbiology program, first-time in college (FTIC), on-campus transfer (OC-TR), and online transfer (ONL-TR) students was performed (Table 1). Transfer students have a higher percentage of underrepresented minorities (URM) in STEM than FTIC students, and ONL-TR have a higher percentage of women than OC-TR or FTIC student populations. Student age increases with transfer status and online track, where ONL-TR students are older than their OC-TR and FTIC peers, and OC-TR students are older than FTIC students. Also, a significantly higher percentage of ONL-TR students (100%) have financial need (estimated using Pell eligibility) than OC-TR (56%) and FTIC (20%) students.

**Table 1:** Characteristics of first-time in college (FTIC), on-campus transfer (OC-TR) and online transfer (ONL-TR) students enrolled in microbiology fall 2018 to spring 2020 semesters (excludes summer)

	**Student group (N)**			**Differences (statistical test)**
	FTIC (318)	OC-TR (188)	ONL-TR (97)	
Sex (% female)	59.7	59.0	73.2	ONL-TR program enrolls more women than FTIC and OC-TR (Fisher’s Exact, p value = 0.03)
URM^1^ (%)	26.7	35.6	37.1	OC-TR and ONL-TR programs enroll more URM students than FTIC (Fisher’s Exact, *p* value = 0.02)
Average Age at Matriculation (SD)	18.4 (±1.2)	22.4 (±4.4)	27.5 (±6.5)	• OC-TR students are older than FTIC (p value <0.001) • ONL-TR students are older than OC-TR and FTIC (p value <0.001) (Kruskal-Wallis test)
Average Semester Course Load (SD)	13 (±3)	11 (±3)	9 (±3)	• OC-TR students take fewer credits than FTIC (p value <0.001) • ONL-TR students take fewer credits than OC-TR and FTIC (p value <0.001) (Kruskal-Wallis test)
Enrolled Part-time (%)	21.8	39.9	79.4	• more ONL-TR students enroll part-time than OC-TR and FTIC (p value <0.001) • more OC-TR students enroll part-time than FTIC (p value <0.001) (Fisher’s Exact)
Average GPA (SD)	3.47 (±0.57)	2.98 (±0.78)	2.83 (±0.87)	FTIC students have higher average GPA than OC-TR and ONL-TR (Kruskal-Wallis test, p value <0.001)
4-year Retention^3^ (%) N	95.5 223	46.3 67	61.9 42	OC-TR and ONL-TR programs have lower retention than FTIC (Fisher’s Exact, p value <0.001)
Financial Need (%)^2^	19.7	55.6	100	• more ONL-TR students have financial need than OC-TR and FTIC (p value <0.001) • more OC-TR students have financial need than FTIC (p value = 0.01) (Fisher’s Exact)

^1^URM, underrepresented minorities in STEM include Black/African American, Hispanic/Latino, American Indian or Alaskan Native, Native Hawaiian or other Pacific Islander

^2^Retention rate is based on student cohorts that matriculated fall 2011 through summer 2014 and either graduated or persisted 4 years after matriculation

^3^Financial Need was determined by students that qualified for Pell federal funding and is used as a proxy for financial need. This is an underestimation of any financial need

Transfer students, both online and on-campus, are more likely to enroll part-time than FTIC students, with the frequency of part-time enrollment greatest amongst online transfer students (79.4%; Table 1). Part-time enrollment is often associated with poorer academic outcomes. ONL-TR students took fewer credits fall and spring semesters than on-campus students (Supplementary Fig. 1) yet performed similarly academically (GPA) to OC-TR students (Table 1), while FTIC students had a higher average GPA than either transfer student group (OC-TR or ONL-TR). Furthermore, transfer students have lower 4-year retention (graduated or persisted) than FTIC students. Although after four years post-transfer, 62% of ONL-TR students were retained versus 46% of OC-TR students (Table 1).

The relationship between part-time enrollment and academic outcomes cannot be so easily simplified given the social and economic differences characteristic of transfer students, which is further nuanced by online modality. Differences in financial need and enrollment patterns would have implications for need-based scholarship eligibility stipulating full-time enrollment.

### Enrollment Status Negatively Impacts Scholarship Eligibility of Online Transfer Students

The microbiology program has a federally funded scholarship program for low-income transfer students, but full-time enrollment is required. A Spring 2019 representative sample was used to assess how part-time enrollment affected the eligibility of students in the transfer cohorts.

For students enrolled in Spring 2019, only 39% (*N* = 44) of the transfer student population met all eligibility criteria to receive the scholarship (light red track, Fig. 1). The percentage of eligible on-campus transfer students was more than two-fold higher than that of online transfer students (51% vs. 22%, *p* value <0.05; Fisher’s Exact Test). The main reason for this discrepancy was full-time enrollment status. Full-time enrollment was the single dominant exclusion criteria for online transfer students compared to not having financial need and/or having a low GPA (gray track, Fig. 1).

Mirroring the general enrollment trends of ONL-TR students, in Spring 2019, online transfer students were 9 times more likely to enroll part-time than on-campus transfer student (p value <0.001, Fisher’s Exact test); 79% of ONL-TR students enrolled part-time compared to 29% of OC-TR students. The full-time enrollment criterion affected a disproportionate number of online transfer students compared to on-campus transfer students. A large percentage (44%, *N* = 20) of online transfer students met all scholarship eligibility criteria except full-time enrollment (purple track, Fig. 1). For comparison, only 6% (*N* = 4) of on-campus transfer students were ineligible only due to part-time enrollment. The part-time otherwise eligible group comprised a higher percentage (75%) of female students than all eligible transfer students (61%) and had a similar percentage of underrepresented minority (URM) students (both 32%). Part-time, otherwise eligible online students had similar annual financial need compared to full-time, eligible online students (Supplementary Table 1).

The full-time criterion also affected retention within the scholarship program. Twelve percent of scholars continuing from the previous fall semester became ineligible because they shifted to part-time enrollment, which accounted for the majority of scholarship attrition. Therefore, the requirement of full-time enrollment disproportionately excludes online transfer students from scholarship opportunities, hence limiting potential resources available to these students to promote their success.

### Predictors of Part-Time Enrollment

Logistic regression was used to identify if demographic characteristics or GPA were associated with full- or part-time status. Knowing that GPA and age were significantly different between the student groups, the student group-to-age and student group-to-GPA interaction terms were included in the model. Sex and URM status did not have a significant impact on part-time enrollment. The effect of age and GPA on part-time enrollment varied across student groups (interaction terms were significant, Table 2; Supplementary Fig. 2). On-campus student groups, whether FTIC or transfer students, were more likely to enroll part-time if they were older or had a lower GPA. For example, a one-year increase in age suggested that an on-campus student was 1.794 times as likely to enroll part-time, while a one unit increase in GPA suggested that a student is 0.3352 times as likely to enroll part-time (Table 2). However, the relationship of age and GPA on part-time enrollment was not consistent for online students.

**Table 2:** Estimated coefficients with odds ratios (OR) and confidence intervals (CI) from logistic regression that estimates how demographic and academic performance predictors impact part-time enrollment. Results for main effects (first 4 rows) and interaction terms (last 4 rows) are provided

Predictors	Estimate	Std. Error	*z* value	p value	OR	CI
Gender - Female	0.01604	0.2179	0.074	0.9413	1.016	0.664–1.56
URM	−0.07402	0.2235	−0.331	0.7404	0.9286	0.596–1.43
Age	0.5845	0.2812	2.079	0.03763*	1.794	1.14–3.34
Mean GPA	−1.093	0.3143	−3.477	0.000507***	0.3352	0.179–0.619
Age:Student group						
Age:OC-TR	−0.3768	0.2851	−1.322	0.1863	0.6861	0.366–1.09
Age:ONL-TR	−0.5801	0.2839	−2.043	0.04106*	0.5599	0.299–0.890
GPA:Student group						
GPA:OC-TR	−0.08178	0.4240	−0.193	0.8471	0.9215	0.394–2.09
GPA:ONL-TR	1.147	0.4255	2.696	0.007019**	3.149	1.35–7.22

Residual deviance: 575.85 on 563 degrees of freedom

Chi-square: 168.8 on 10 DF, p value: 4.98e-31

Statistical significance is indicated by *, *p* < 0.05; **, *p* < 0.01; ***, *p* < 0.001

Model formula: part-time ~ Gender + URM + Age + GPA + Age:StudentGroup + GPA:StudentGroup

To further explore this relationship and better understand the model, predicted probabilities for part-time enrollment were calculated at varying values of age (Fig. 2A) or GPA (Fig. 2B) for each student group while holding all other variables constant. For both on-campus groups, FTIC and OC-TR, the predicted probability of enrolling part-time is positively associated with age and negatively associated with GPA. In contrast, for online transfer students, the predicted probability of enrolling part-time does not vary greatly with age or GPA and remains between 0.7 and 0.8 at various values of age and GPA. Thus, age and GPA have stronger effects on part-time enrollment status for on-campus (FTIC or OC-TR) than online (ONL-TR) students.

### Part-Time Enrollment and Academic Outcomes – Online to on-Campus Transfer Student Comparison

Despite online transfer (ONL-TR) students enrolling part-time at higher levels than on-campus transfer (OC-TR) students, they still had comparable, if not better, graduation and overall retention rates after 4 years than OC-TR students (Table 1). This observation led to a more direct comparison of ONL-TR and OC-TR student academic outcomes.

**Fig. 3:**
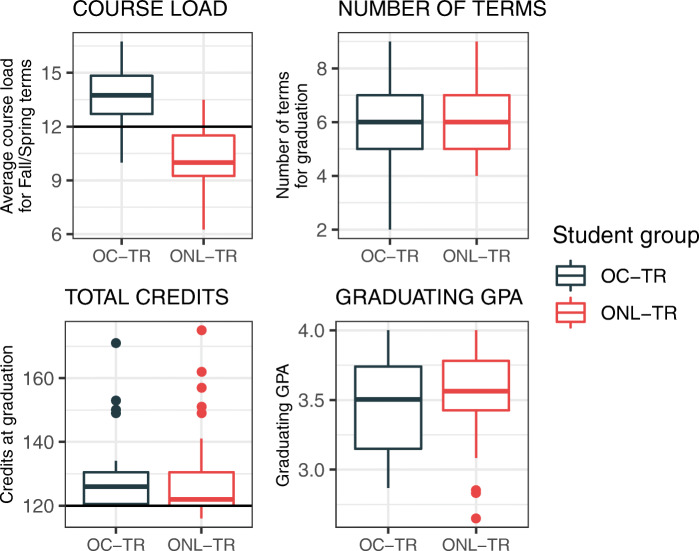
A comparison of OC-TR and ONL-TR students that graduate within 4 years of transferring to microbiology with their AA. COURSE LOAD is the average per student credit load for fall and spring semesters; full-time (12 credits) is annotated with a horizontal line. NUMBER OF TERMS is the total number of terms a student enrolled before graduating, including summer terms. TRANSIENT CREDITS is the number of credits acquired at an outside institution while enrolled at UF. GRADUATING GPA is the UF GPA at the time of graduation

Online and on-campus transfer students that graduate within 4 years (*N* = 22 and *N* = 31, respectively; 2011 to 2014 transfer cohorts) showed that successful online transfer students rarely enrolled full-time, defined as 12 or more credits per semester (median is 10 credits), yet still managed to graduate within the same timeframe and with similar GPAs to on-campus transfer students (Fig. 3). Yet, online and on-campus transfer students graduate with similar total credits (approximately 130, median). Online transfer students compensated part-time enrollment with some transient course work, credits acquired from an outside institution. Eighty-six percent of ONL-TR students that graduated within 4 years received some transient credit, median was 8 transient credit hours. This observation is in contrast to on-campus transfer students very rarely taking any transient courses (3%). Therefore, part-time enrollment of online transfer students does not appear to hinder graduation/retention compared to on-campus transfer students.

### Student Insights to Enrollment Behavior

A survey was administered fall 2019 to all microbiology majors with the intention of understanding undergraduates’ motivations for enrolling full- versus part-time. There were 71 respondents, 15.**2**% of microbiology majors enrolled Fall 2019, each student group represented proportionally. Demographic characteristics (i.e. sex, race, age) were representative of the full enrollment cohort (Supplementary Table 2).

#### Difficulty of Enrolling Full-Time

**Fig. 4:**
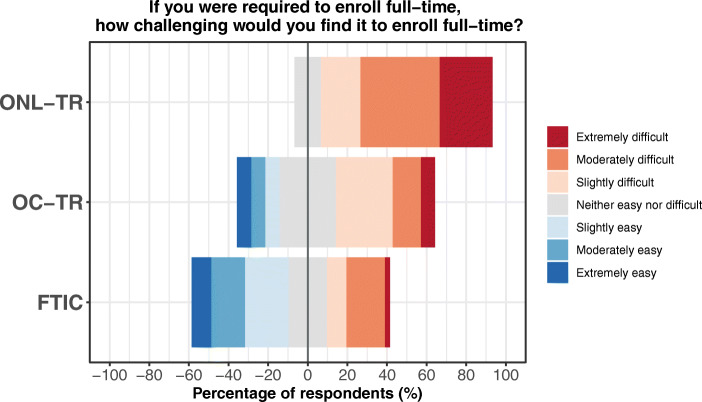
Responses to a Likert Scale question about difficulty of enrolling full-time. Responses ranged from extremely easy to extremely difficult and each response was translated to a numerical score. Transfer students expressed greater challenge in enrolling full-time, and online transfer students (ONL-TR) were more likely to find full-time enrollment difficult than on-campus transfer (OC-TR) and first-time in college (FTIC) students (*p* value <0.01, Kruskal-Wallis test)

When identifying respondent’s enrollment behavior, 88% and 100% of FTIC and OC-TR respondents, respectively, said they always enroll full-time fall/spring semesters, compared to just 40% of ONL-TR respondents. When asked about the challenge of enrolling full-time if required to do so, 87% of ONL-TR and 50% of OC-TR students reported difficulty in full-time enrollment compared with 31% of FTIC students (Fig. 4; *p* value <0.01, Kruskal-Wallis test). Therefore, not only are online transfer students less likely to enroll full-time, they are more likely to find it difficult to enroll full-time if required to do so.

#### Work and Family Responsibilities Motivate Part-Time Enrollment for Online Transfer Students

The motivations and challenges of enrollment status differed among on-campus and online transfer students. Online transfer respondents indicated that having children and being employed were the primary challenges to enrolling full-time, whereas maintaining financial aid eligibility was a determining factor for OC-TR students in enrolling full-time. Forty percent of ONL-TR respondents have children and strongly agreed that this factor prevented them from enrolling full-time. In contrast, no OC-TR respondents and less than 5 (the minimum reportable threshold) FTIC respondents have children. Similarly, 67% of ONL-TR respondents work full-time (40 h or more per week) and agreed that work prevented them from enrolling full-time; less than 5 (the minimum reportable threshold) FTIC and OC-TR respondents worked full-time. While approximately half of FTIC and OC-TR respondents were employed part-time (less than 40 h per week). Less than half of these respondents agreed that part-time employment prevented them from enrolling full-time. Therefore, with greater family and work responsibilities than their on-campus peers, online transfer respondents agreed that part-time enrollment allowed them to focus on demanding courses (80%) and helped them achieve higher academic performance (67%).

#### Student Voices - Thematic Analysis

Thematic analysis of a free-response question asking students to “briefly discuss factors that influence your decision to enroll full-time vs. part-time” showed that, regardless of student group, the main factors affecting enrollment decisions were Funding, Perceived Advantages/Disadvantages, and Personal Reasons. The high coding density observed across data from several participants showed that the three themes are highly interrelated. Within the Funding theme, scholarships and grants (with their associated requirements) were frequently identified as key factors that both allow and force students to enroll full-time. In the words of one participant, “I have to enroll full time for my scholarships to apply but it makes classes extremely difficult for me and I believe I would have a better educational experience if I were able to do part-time.” Admission requirements of some graduate/professional STEM programs also factored in the enrollment decisions of a few participants.

Many participants also considered that enrolling part-time would benefit their academic performance (Perceived Advantages/Disadvantages theme). However, they still enrolled full-time to meet the requirements of their funding: “The primary reason that I am enrolled full-time is to meet the requirements for the scholarship. Otherwise, I would be enrolled part-time because having more time to focus on individual classes allows me to retain the material much better.” In addition to performance, time management and time-to-graduation were also sub-themes dealing with perceived advantages and/or disadvantages that factor into enrollment decisions.

The Personal Reasons theme encompassed a variety of aspects including cultural considerations (“Growing up, I knew that after I graduated high school I’d matriculate into university to continue my education as a full-time student.”), health status (“Health issues - either mine or those of members of my family - have at times made it difficult to handle full-time.”), and economic responsibilities (“I am an online student because I have a full-time job, I am expecting a baby and all that drives me crazy, the requirement of full-time enrollment is more than difficult to achieve a good GPA.”).

#### Financial Aid Motivations of Enrollment Status Differ by Student Group

All on-campus transfer respondents indicated that they enroll full-time, and all (100%) strongly agreed that maintaining scholarship and/or financial aid eligibility influenced their decision to enroll full-time. Thus, financial aid and scholarship eligibility is a primary factor influencing enrollment behavior for OC-TR students. Yet, half of on-campus transfer respondents would find mandatory full-time enrollment difficult (Fig. 4). In contrast, FTIC respondents indicated similar enrollment behavior (88% always enroll full-time) but less often strongly agreed that financial aid eligibility (69% compared to 100% for OC-TR respondents) influenced their decision to enroll full-time. Furthermore, 57% of OC-TR respondents were Pell eligible (a proxy for determining financial need), compared to 24% of FTIC respondents, suggesting OC-TR students feel obligated to enroll full-time for financial reasons.

Online transfer students less often strongly agreed (53% compared to 100% of OC-TR respondents) that financial aid and scholarship eligibility influences their decision to enroll full-time, but less than half (40%) of this group indicated that they always enroll full-time (compared to 100% of OC-TR respondents). A similar percentage of ONL-TR students (60% compared to 57% of OC-TR respondents) were Pell eligible but were more likely to be employed full-time. This result indicates that both online and on-campus transfer groups find financial aid and scholarship eligibility an important factor in their decision to enroll full- versus part-time, but OC-TR students are more reliant on financial aid and scholarships, whose eligibility criteria is more likely to dictate enrollment behavior. Whereas ONL-TR respondents are more likely to meet financial responsibilities through employment rather than financial aid and/or scholarships.

## Discussion

This study finds that microbiology transfer students are more likely to be underrepresented minority students in STEM, women, older, low-income, parents, enrolled part-time, employed full-time, and to have a lower GPA. These characteristics are even more pronounced among microbiology transfer students who are in the online track in which 79% enroll part-time. These findings agree with other studies of transfer students (Radwin et al., 2018). While scholarships would be helpful interventions for transfer students, the full-time enrollment stipulations tied to these programs are a real barrier for transfer students. This is especially true for online transfer students as they are more likely to enroll part-time. This result was demonstrated by a deeper analysis into the Spring 2019 semester indicating that 44% of the online transfer students who satisfied all eligibility requirements, including the GPA threshold, were ineligible to receive a scholarship solely due to their part-time enrollment status.

Many of the policies that push full-time enrollment stem from the idea that full-time enrollment leads to greater retention of students (Lee, 2018), which has been observed in traditional on-campus programs. Altogether, part-time enrollment has generally been seen as a risk factor for attrition (Bean & Metzner, 1985; O'Toole et al., 2003) and associated with lower academic aspirations and degree achievement (Chen, 2007). However, these findings were restricted to on-campus students and did not distinguish students at 4-year institutions or intent to earn a BS degree. Academic and career motivation is a strong factor influencing a student’s success and degree attainment. Beyond graduation, there is little data tracking long-term outcomes of students, though evidence suggests that online and face-to-face delivery of lecture courses had similar post-graduate degree outcomes (Ardissone et al., 2020). The current study specifically focuses on online transfer students who intend to obtain a B.S. degree, which implies increased motivation relative to the students in which findings of part-time enrollment as a risk factor for student attrition are based. High motivation of this student group is indicated by higher retention (Table 1) and graduating GPA (Fig. 3) of ONL-TR compared to OC-TR students.

In the microbiology online transfer track, even though a majority of the online transfer cohort enrolls part-time, the retention of the cohort is higher than the on-campus transfer cohort that enrolls nearly exclusively full-time. Looking more closely at the students who successfully graduated from either the online or on-campus transfer track, the graduates from the online transfer track enrolled part-time and completed with higher GPAs than their on-campus transfer counterparts. Despite part-time enrollment, online transfer students graduated with a similar number of credits as on-campus transfer students and were achieving this by transferring in more credits, earned pre-transfer and/or transiently from other institutions while concurrently enrolled at the bachelors-granting university. This program demonstrates that part-time students can be as or more successful than full-time students and employ creative strategies for meeting degree requirements.

Logistic regression analysis indicated that part-time enrollment was associated with age and GPA, but that this relationship differed for students in the online transfer track. For both on-campus groups, non-transfer and transfers, the predicted probability of enrolling part-time is positively associated with age, meaning that the older a student is, the more likely they are to be enrolled part-time. This is in agreement with a national report indicating that on-campus, part-time students are more likely to be older (Chen, 2007). The opposite relationship was observed for GPA in that the lower the GPA, the more likely the student was enrolled part-time. This negative association between GPA and enrollment status has been observed in other studies and is one rationale for pushing policies that encourage full-time enrollment (Lee, 2018). In contrast, for online transfer students, the predicted probability of enrolling part-time does not vary with age or GPA and remains steady. Regardless of age or GPA, an online transfer student is 70–80% likely to be enrolled part-time. The predicted probabilities depicted in Fig. 2 illustrates how the on-campus and online cohorts do not fit the same model of behavior, and scholarship policies rarely consider such student group differences.

Many eligible online transfer students were aware that enrolling for 12 credits (full-time) instead of 10 credits (part-time) would make them eligible for substantial scholarships. Yet, few were able or willing to enroll full-time. The logistic modeling of institutional data presented two models of enrollment patterns for two different populations of students, but a qualitative survey addressed the why of part-time enrollment. The survey revealed that two-thirds of the online transfer students work at least 40 h per week and half of them have children. This result stands in sharp contrast with the on-campus students in which only 2–5% of respondents reported working full-time or having children. As such, online transfer respondents indicated that work and parenting were two factors that influenced their decision to enroll part-time. Nearly all of the online transfer respondents expressed that enrolling full-time would be challenging and unlike the on-campus transfer students, none of the online transfer students indicated that enrolling full-time would be easy.

Students that are employed full-time, parents, or married are more likely to enroll part-time and engage in online postsecondary education due to lower opportunity costs compared to on-campus degree programs (Ortagus, 2017). Therefore, part-time enrollment is a common characteristic of nontraditional, online students in which there is no convincing evidence that it hinders their academic success and should thus not be an exclusion criterion for scholastic opportunity, be it scholarships or other academic resources. Given the increasingly heterogeneous nature of students in higher education, a more holistic consideration of their characteristics and aspirations is warranted in designing and determining solutions and interventions aimed at ensuring student success. For example, by exploring the pathways and needs of different students, it can be concluded that a needs-based scholarship with a full-time requirement is a feasible and promising intervention for the on-campus transfer students, but is not as feasible for the online transfer students of this study.

Whether nontraditional, transfer, online or a myriad of other characteristics that contribute to the vast heterogeneity of today’s undergraduate students, it is clear that trends concerning student success with a specific characteristic cannot be applied indiscriminately to all students who share that characteristic. For example, research pertaining to online education usually fails to differentiate the residential student taking one online course and the nontraditional learner enrolled in a fully online program, so findings are not generalizable beyond individual courses or programs and are thus of limited value for administrators and policymakers (Lack, 2013). This study is specifically concerned with transfer students in a hybrid online STEM program who possess multiple nontraditional characteristics. These findings contribute to a growing body of knowledge and awareness that part-time enrollment as a prevailing characteristic of nontraditional, online students does not imply increased likelihood of attrition as proposed by conceptual models that originated in an era prior to online education.

This point is particularly important in current education policy, in which government resource allocations are based on full-time student enrollment (Welton et al., 2020). As reviewed in Grabowski et al. (2016), students who enroll less than full-time do not qualify for as many types of tuition assistance and the financial burden of attending college is much greater than compared to full-time students. Education policies requiring full-time enrollment are insensitive to the over 7 million part-time students nationwide enrolled at a postsecondary institution, resulting in a rising voice to extend resources to part-time students (Goldrick-Rab et al., 2016), who are likely to benefit the most. While the majority of students at 2-year institutions enroll part-time, these issues extend to students attempting to bridge the 2 + 2 transfer gap and pursue a bachelor’s degree at a 4-year institution and should not be overlooked. This point is reflected in OC-TR students enrolling full-time despite expressing that full-time enrollment is very challenging. While scholarships would provide much needed financial support to both online and on-campus transfer students (100% and 56% Pell eligible, respectively), OC-TR students expressed an obligation to enroll full-time for financial reasons, at times to the detriment of academic and learning outcomes. Whereas ONL-TR students utilized full-time employment as a means to meet financial responsibilities rather than financial aid and/or scholarships and be subject to the eligibility constraints imposed.

National institutions’ acknowledgement of part-time students is encouraging including the Integrated Postsecondary Education Data System’s (IPEDS) categorization of students by first-time enrollment *and* full/part-time enrollment status. Indeed, the federally funded scholarship program that led to this work reduced the full-time requirement to half-time in spring 2020, which is an exciting and welcome development. However, this change may not be permanent, and the nation’s students are eagerly awaiting policy action at a higher level. With enrollment of part-time students projected to outpace full-time students by 2027 (5% compared to 2% growth, respectively) (Hussar & Bailey, 2019), it is critical that education policies and programs predicated on full-time enrollment status be reconsidered and adjusted accordingly. The increased growth of part-time compared to full-time enrollment, may become even wider if the economic effects of the current COVID-19 global health pandemic linger over the coming years.

Educational policies pushing full-time enrollment is unnecessarily selective against nontraditional students, especially online students. Full-time is not a warranted criterion for intervention strategies and does not equate with student success. For example, OC-TR students struggle to enroll full-time but feel financially obligated to do so, which comes at the expense of academic achievement (lower GPA and retention compared to FTIC). Students have their own motivations for enrolling full-time. With the reduced criterion of half-time enrollment for this scholarship, it is worth noting that less than 10% of scholarship recipients reduced their enrollment to half-time for the subsequent fall semester. The change in required course load increased the number of eligible scholarship recipients rather than promoting part-time enrollment. Enrollment status should be a decision students make for primarily academic and knowledge gains. While full-time enrollment may be in the best interest of some, it should be left to the student to enroll to the extent they feel best suits their needs, which are varied and complex, without feeling the undue pressure of compromising financial support.

Accelerated by the adoption of online education, the face of higher education continues to evolve to be more inclusive and accessible. While there is undoubtedly much work to be done, this effort needs to happen on multiple fronts, including policy change. The typical full-time enrollment criteria attached to many scholarship programs, while well-intentioned, have created considerable barriers for increasingly diverse student populations they are meant to serve. This diversity extends in ways beyond race, ethnicity and gender to include lifestyle, family circumstances, employment, income, and learning modalities. Hence while programs are working diligently to increase their flexibility in order to extend their reach and accessibility, they are subjected to roadblocks at a higher level and can only be successful if institutional policies adapt to meet the increased demand for flexible education. Of course, increased flexibility should not sacrifice educational quality and rigor, which is a major and essential concern. While progress is slow, but persistent, one should remain realistically optimistic that STEM higher education and policy can and will evolve to meet the demands of a changing society.

## Supplementary Information

ESM 1 (DOCX 124 kb)

## Data Availability

Requests for the data should be directed to the corresponding author.
