# Supplementary material for: The Need for Equitable Scholarship Criteria for Part-Time Students
Source: Innov High Educ. 2021 Apr 19;46(4):461–79. doi: 10.1007/s10755-021-09549-7 (PMC8054119; doi:10.1007/s10755-021-09549-7)
Supplement: Supplementary file 1 — (DOCX 124 kb) [file 10755_2021_9549_MOESM1_ESM.docx]

Supplementary Material

# Supplementary Figures and Tables

## Supplementary Figures

**Supplementary Figure 1**. Student average course load from Fall 2018 to Spring 2020 are represented by student groups. Full-time enrollment for Fall/Spring semesters is defined as 12 credits (horizontal line). FTIC students are students who were admitted as first time in college students (non-transfers) and are on-campus. On-campus transfer students are denoted as OC-TR, and ONL-TR represents students in the online 2+2 transfer track.

**Supplementary Figure 2.** Boxplots of age (A) and GPA (B) for full-time (FT) and part-time (PT) students across student groups. FTIC, first-time in college; OC-TR, on-campus transfer; and ONL-TR, online transfer.

## Supplementary Tables

**Supplementary Table 1.** Characteristics of scholarship eligible on-campus, eligible online and PT otherwise eligible online transfer students enrolled Spring 2019. Transfer students ineligible due to additional scholarship criteria are not presented.

|  | **Online (45)** | | **On-Campus (67)** |
| --- | --- | --- | --- |
|  | PT otherwise eligible  (N=20, 44%) | FT eligible  (N=10, 22%) | FT eligible  (N=34, 51%) |
| Course Load  (median (range)) | 8 (3:11) | 13 (12:18) | 14 (12:19) |
| UF GPA  (median (range)) | 3.0 (2.5:3.6) | 3.9 (2.75:4.0) | 3.3 (2.5:4.0) |
| Annual Financial Need  (median (range)) | $10,017  ($2,617:$16,709) | $10,019  ($1,112:$16,895) | $15,070  ($365:$47,716) |
| Percent Female  (% (N))  (excluded not reported) | 75% (15) | 56% (5) | 65% (22) |
| Percent URM  (% (N))  (excluded not reported) | 32% (6) | * | 30% (10) |

*Percentage did not meet minimum reporting criteria of 5 students

**Supplementary Table 2.** Demographic characteristics of survey respondents by group.

| **Student group** | **FTIC (42)** | **OC-TR (14)** | **ONL-TR (15)** |
| --- | --- | --- | --- |
| Sex (% female) | 76 | 86 | 53 |
| URM^1^ (%) | 17 | 43 | 40 |
| Older than 24 years (%) | * | * | 67 |
| Employed full-time (≥40 hours/week) (%) | * | 0 | 67 |
| Have children | * | 0 | 47 |
| Parent education is 4-year degree or higher (%) | 67 | 64 | * |
| Enrolled part-time (%) | * | 0 | 40 |
| *Perceived Enrollment Behavior*  Always enroll FT  Prefer to enroll FT, but sometimes PT  Prefer to enroll PT, but sometimes FT  Always enroll PT | 88  *  *  * | 100  0  0  0 | 40  33  *  * |

^1^URM, underrepresented minorities in STEM include Black/African American, Hispanic/Latino, American Indian or Alaskan Native, Native Hawaiian or other Pacific Islander.

*Percentage did not meet minimum reporting criteria of 5 students
